# Speech‐Based Detection of Alzheimer's Disease: Leveraging Spectral Contrast and Pitch Variability as Potential Diagnostic Markers

**DOI:** 10.1002/alz70856_106182

**Published:** 2026-01-11

**Authors:** Hamed Azami, Saturnino Luz, Sanjeev Kumar

**Affiliations:** ^1^ Centre for Addiction and Mental Health, Toronto, ON, Canada; ^2^ University of Toronto, Toronto, ON, Canada; ^3^ Toronto Dementia Research Alliance, Toronto, ON, Canada; ^4^ Usher Institute, Edinburgh Medical School, The University of Edinburgh, Edinburgh, Edinburgh, United Kingdom; ^5^ Department of Psychiatry, University of Toronto, Toronto, ON, Canada

## Abstract

**Background:**

Alzheimer's disease (AD) profoundly affects motor control and cognitive functions, often resulting in impaired speech characteristics such as vocal clarity, emotional expressiveness, and prosodic richness. To detect such abnormalities, we examine the role of three specific acoustic features to differentiate participants with AD from healthy controls (HC) and study the association between these acoustic features and global cognition.

**Methods:**

Speech data from 237 participants (115 HC, 110 AD) in the ADReSS‐M dataset, collected during the “Cookie Theft” picture description task, were analyzed. This dataset has been matched for age and gender by propensity score to prevent bias. The HC group averaged 66.4 years (SD: 6.64), and the AD group averaged 69.4 years (SD: 6.92), with significantly lower Mini‐Mental State Examination (MMSE) scores in AD (AD: 17.9; HC: 29.0). Features quantifying vocal clarity, articulatory precision (spectral contrast), vocal tone (pitch mean), and prosodic variability (pitch standard deviation) were extracted. Group differences in these features were assessed using t‐tests, and Pearson correlation analyses were conducted to examine associations between acoustic measures and MMSE scores.

**Results:**

There were significant differences between AD and HC groups for spectral contrast (t(235) = 4.26, *p* <0.0001), pitch mean (t(235) = 3.54, *p* = 0.0005), and pitch standard deviation (t(235) = 3.62, *p* = 0.0004). Cohen's d values for these features ranged from ‐0.5 to ‐0.6, indicating medium effect sizes, with lower values observed in the AD group. We also found a significant correlation (*p* <0.01) between MMSE scores and each of the features (Pearson's r = 0.22 for pitch mean, 0.23 for pitch standard deviation, and 0.25 for spectral contrast).

**Conclusions:**

This preliminary study highlights the physiological basis of altered speech patterns in AD and their diagnostic relevance. Future work will focus on refining preprocessing algorithms and incorporating advanced feature extraction methods to enhance the effect sizes and correlation for AD detection and cognitive assessment.

